# Identification of S-locus F-box Protein Sequences in Diploid Potato, *Solanum okadae*, via Degenerate PCR

**DOI:** 10.21769/BioProtoc.5328

**Published:** 2025-06-05

**Authors:** Amar Hundare, Ranjan Swarup, Timothy P. Robbins

**Affiliations:** Division of Plant and Crop Science, School of Biosciences, University of Nottingham, Sutton Bonington Campus, Loughborough, UK

**Keywords:** Degenerate PCR, Orthologs, Self-incompatibility, SLF, Solanaceae, *Solanum okadae*

## Abstract

In many plant species, self-incompatibility (SI) is a mechanism that inhibits inbreeding. SI is gametophytic in the Solanaceae, with specificity determined by S-ribonucleases (S-RNases) in the pistil and S-locus F-box proteins (SLFs) in the pollen. The role of these proteins has been studied extensively in the Solanaceae, often using *Petunia* as a model. Using degenerate PCR and Sanger sequencing, this protocol identified three SLF sequences from self-incompatible diploid potato (*Solanum okadae*). While SLFs are well-characterized in model species like *Petunia*, there is limited sequence data and no standardized protocols for identifying SLFs in non-model species such as *S. okadae*, hindering broader insights into SI across the Solanaceae. This protocol fills that gap by using degenerate PCR and Sanger sequencing with primers designed from conserved *Petunia* SLF regions to identify SLF sequences in *S. okadae*. SLF sequences from 10 distinct Solanaceae members sharing maximum identity with the S2-haplotype of *Petunia* were used to design two pairs of primers targeting different regions of the target sequence. PCR amplification using designed degenerate primers yielded amplicons that were directly sequenced and joined together to get the partial SLF sequence. It was observed that the *S. okadae* shared an orthologous relation with the *Petunia* SLF according to the phylogenetic analysis. These SLFs could be used in future SI breakdown experiments via the competitive interaction route. This protocol, including the primer design, is novel for detecting SLF sequences in *S. okadae*.

Key features

• This protocol is applicable when the exact nucleotide sequence of the target DNA is not known but can be deduced from an amino acid sequence.

• Straightforward, cost-effective, and can be used to find “new” genes or gene families.

• Guidelines and a systematic approach for designing degenerate primers, along with a framework for annotating and comparing SLF genes within the Solanaceae family.

## Background

Many plant species use self-incompatibility (SI) to prevent inbreeding. SI is governed by the polymorphic S-locus, which contains a single pistil-specific S-RNase gene along with a collection of pollen-specific S-locus F-box (SLF) genes. The developing pollen tube absorbs cytotoxic S-RNases from the pistil; however, only those S-RNases that correspond to the pollen's S-haplotype impede pollen tube growth. The collaborative non-self-recognition model [1] posits that each SLF protein functions as an E3 ubiquitin ligase subunit, facilitating the ubiquitination and subsequent degradation of its interacting non-self-S-RNase(s). The complete suite of SLF proteins must collaborate to detoxify all non-self-S-RNases, facilitating cross-compatible pollination and promoting outcrossing. The expression of an SLF from a different S-haplotype in pollen protects the pollen from its own S-RNase, thereby eliminating incompatibility, a process referred to as competitive interaction [2]. A previous study [3] found that *Petunia* S-RNases can be detoxified by SLFs from various taxa in 22 of 26 cases.

The identification of novel members of gene families through polymerase chain reaction (PCR) utilizing degenerate primers has often been perceived as more of an artistic endeavor than a scientific one. This perception has resulted in reluctance among researchers in creating methodologies that adequately tackle the essential factors related to experimental design, along with the lack of a detailed protocol for its implementation. Most applications of PCR rely on the precise design of primers that correspond to a designated target sequence. In the absence of adequate genome sequence data, degenerate consensus primers derived from reverse-translated sequences of highly conserved amino acid regions are commonly employed to isolate homologous sequences of specific gene family members in various organisms. Regions are generated through multiple amino acid alignments or synthesis of protein blocks primarily based on the sequences of distantly related species [4]. The present study employed a similar approach to identify three putative SLF sequences in *S. okadae*, which could be used to identify other SLFs in other *Solanum* species. These might be employed in future research to break down the SI by competitive interaction, an alternative to S-RNase knockout.

## Materials and reagents

**Biological materials**

1. Seeds of *Solanum okadae* (accession: OKA 7129) obtained from The James Hutton Institute (Invergowrie, Scotland) were germinated and grown in a glasshouse environment at 22 °C ± 2 °C with a 16 h photoperiod. The following protocol uses young leaves from 2-week-old plants

**Reagents**

1. GenElute^TM^ Plant Genomic DNA Miniprep kit (Sigma-Aldrich, catalog number: G2N70-1KT)

2. MyTaq Extract-PCR kit (Bioline, catalog number: BIO-21126)

3. GeneJET Gel Extraction kit (Thermo Scientific, catalog number: K0691)

4. Agarose (Sigma-Aldrich, catalog number: A9539-500G)

5. UltraPure^TM^ TBE buffer, 10× (Invitrogen, catalog number: 15581-044)

6. Ethidium bromide (EtBr) (Fisher Bioreagents, catalog number: BP1302-10)

7. DNA HyperLadder 1 kb (Bioline, catalog number: BIO-33053)

8. 5× DNA loading buffer blue (Bioline, catalog number: BIO-37045)

**Solutions**

1. 1× TBE buffer (working) (see Recipes)

2. 0.8% (w/v) agarose gel with EtBr (see Recipes)

**Recipes**

**1. 1**× **TBE buffer (working)**

Mix 100 mL of 10× UltraPure^TM^ TBE buffer thoroughly with 900 mL of distilled water.

**2. 0.8% (w/v) agarose gel with EtBr**

Add 0.8 g of agarose per 100 mL of 1× TBE buffer and heat until agarose is dissolved. Let it cool for 5 min. Add 5 μL of EtBr just before pouring the gel in the electrophoresis tray on the lab bench.

**Laboratory supplies**

1. Pipette tips (10, 100, 1,000 μL) (Thermo Scientific)

2. 1.5 mL Eppendorf tubes (Sarstedt, catalog number: 72.690)

3. PCR 8-tube strips with individual attached caps (Greiner bio-one, catalog number: 608281)

## Equipment

1. Electric water bath (Grant instruments Ltd., Cambridge, model: BAT3312)

2. Mortar and pestle (KPM, Berlin, catalog number: 39304000)

3. Analog vortex mixer (VWR International, catalog number: OHAU30392141)

4. Digital balance (A&D Company Limited, model: HF4000)

5. Centrifuge (Thermo Scientific, model: Pico 21)

6. Forceps (Bochem^TM^ Stainless Steel Sharp Tip Forceps, catalog number: 1133)

7. Scalpel (carbon steel surgical blade, Swann-Morton)

8. Automated thermal cycler (Applied Biosystems, model: GeneAmp 9700)

9. Spectrophotometer (NanoDrop Technologies, model: ND-1000 Spectrophotometer)

10. Gel casting tray & power pack (Bio-Rad, catalog number: 1640302)

11. UV transilluminator crosslinkers (Syngene, catalog number: 12874028)

## Software and datasets

1. NCBI BLAST (http://blast.ncbi.nlm.nih.gov/Blast.cgi)

2. EMBOSS Backtranseq (https://www.ebi.ac.uk/Tools/st/emboss_backtranseq/)

3. SnapGene^®^ Viewer version 6.0.2 (GSL Biotech; available at snapgene.com)

4. ClustalΩ (Sievers F & Higgins DG, 2014) (https://www.ebi.ac.uk/Tools/msa/clustalo/)

5. DNA reverse complement tool (https://www.bioinformatics.org/sms/rev_comp.html)

6. Expasy translation tool (https://web.expasy.org/translate/)

## Procedure

**A. DNA extraction**

1. Using a mortar and pestle, grind ~500 mg of plant leaves into a fine powder in liquid nitrogen. Weigh 100 mg of the powder using a digital balance and transfer to an Eppendorf tube. For immediate usage, place the sample on ice; alternatively, freeze at -70 °C.

2. Using the GenElute™ Plant Genomic DNA Miniprep kit, extract genomic DNA from a powdered leaf sample according to the manufacturer's instructions.

3.Using a NanoDrop spectrophotometer, determine the concentration and purity of the plant DNA. The absorbance ratio of 260 nm to 280 nm (A_260_/A_280_) should be ~1.8. A ratio < 1.8 suggests protein contamination, whereas ratios > 2.0 indicate RNA contamination.

4. For short-term storage (1–4 weeks), keep the DNA at 2–8 °C. To preserve the DNA for longer periods (1–2 years), store at -20 °C. Avoid repeated freezing and thawing, which might result in DNA strand breakage.

**B. Degenerate primer design**

**Figure 1.**
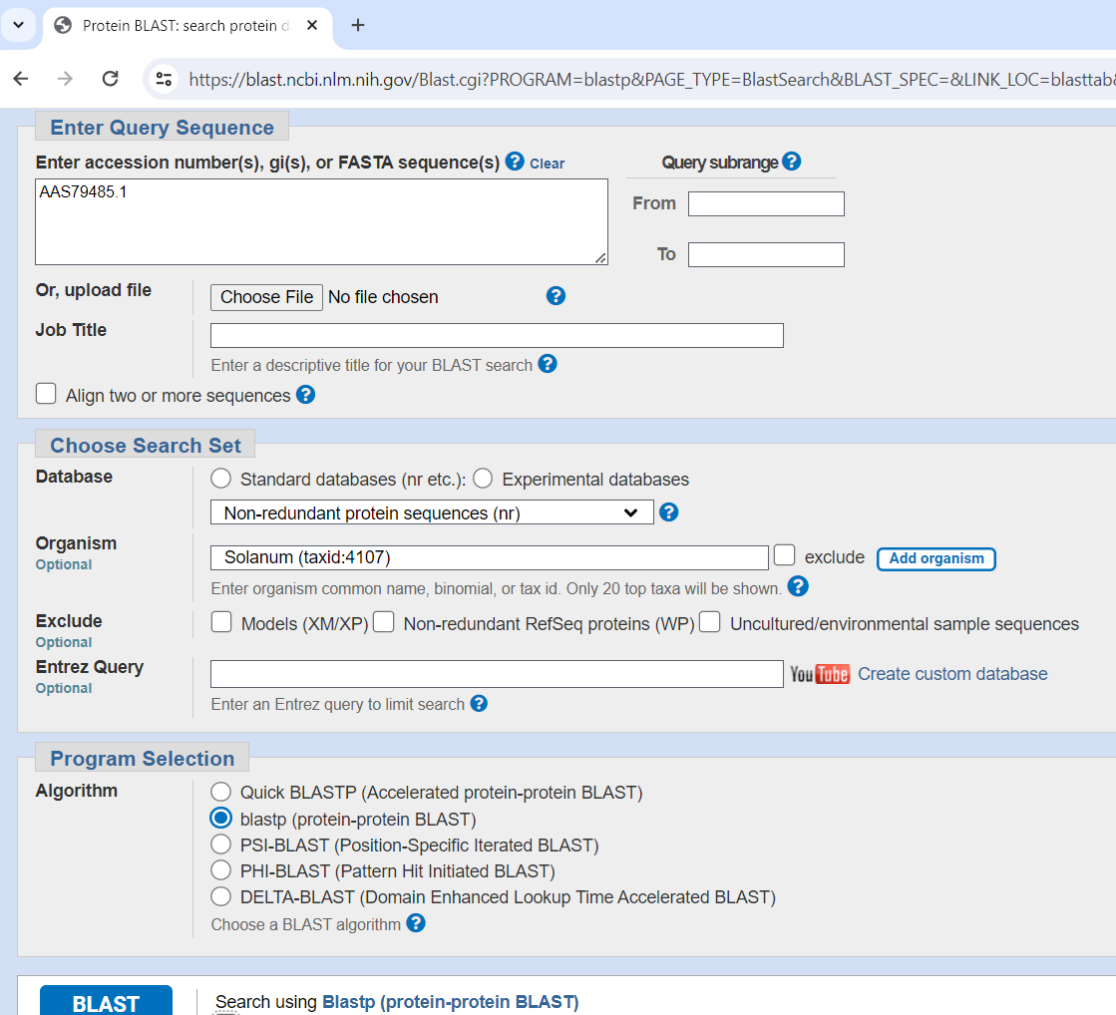
Protein-protein BLAST alignment with known (S2-SLF1) *Petunia* SLF sequence

**Figure 2.**
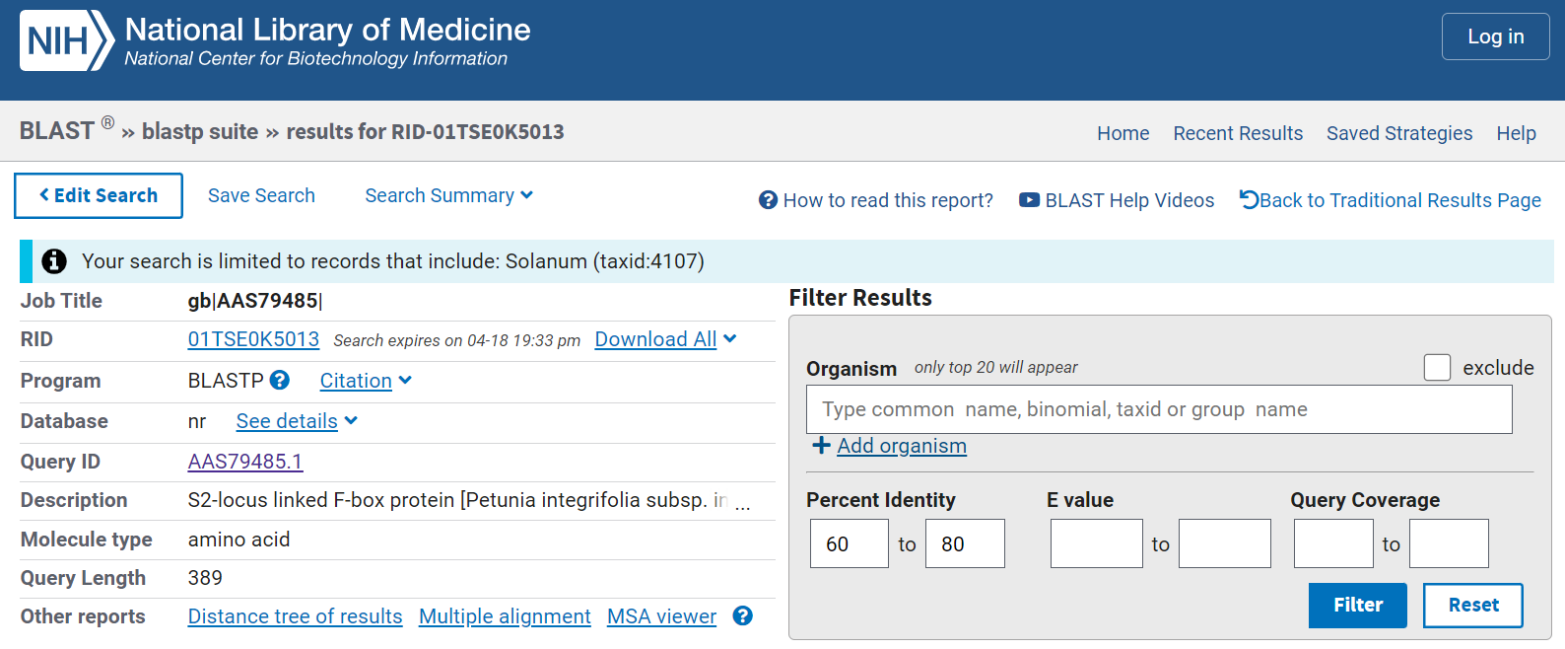
BLAST *Result* window showing the *Percent identity* filter and *Multiple alignment* option

1. Retrieve the amino acid sequence for a specific, known SLF of *Petunia integrifolia subsp. inflata* from NCBI protein database ( https://www.ncbi.nlm.nih.gov/protein ) or UniProt database (https://www.uniprot.org/), for example, the S2-locus linked F-box protein (accession: AAS79485) (Figure 1).

2. Perform a protein-protein BLAST alignment with the known *Petunia* SLF sequence as a query against the non-redundant protein sequence (nr) database and *Solanum* (taxid: 4107) as the organism.

3. On the *Result* window, filter the results by *Percent identity* (between 60% and 80%) and click on *Multiple Alignment* (Figure 2).

*Note: SLFs are fast-evolving genes, especially within the S-locus, so lower identity does not automatically mean non-orthology. Functional domains and conserved motifs may still be preserved even with 60% overall identity.*

**Figure 3.**
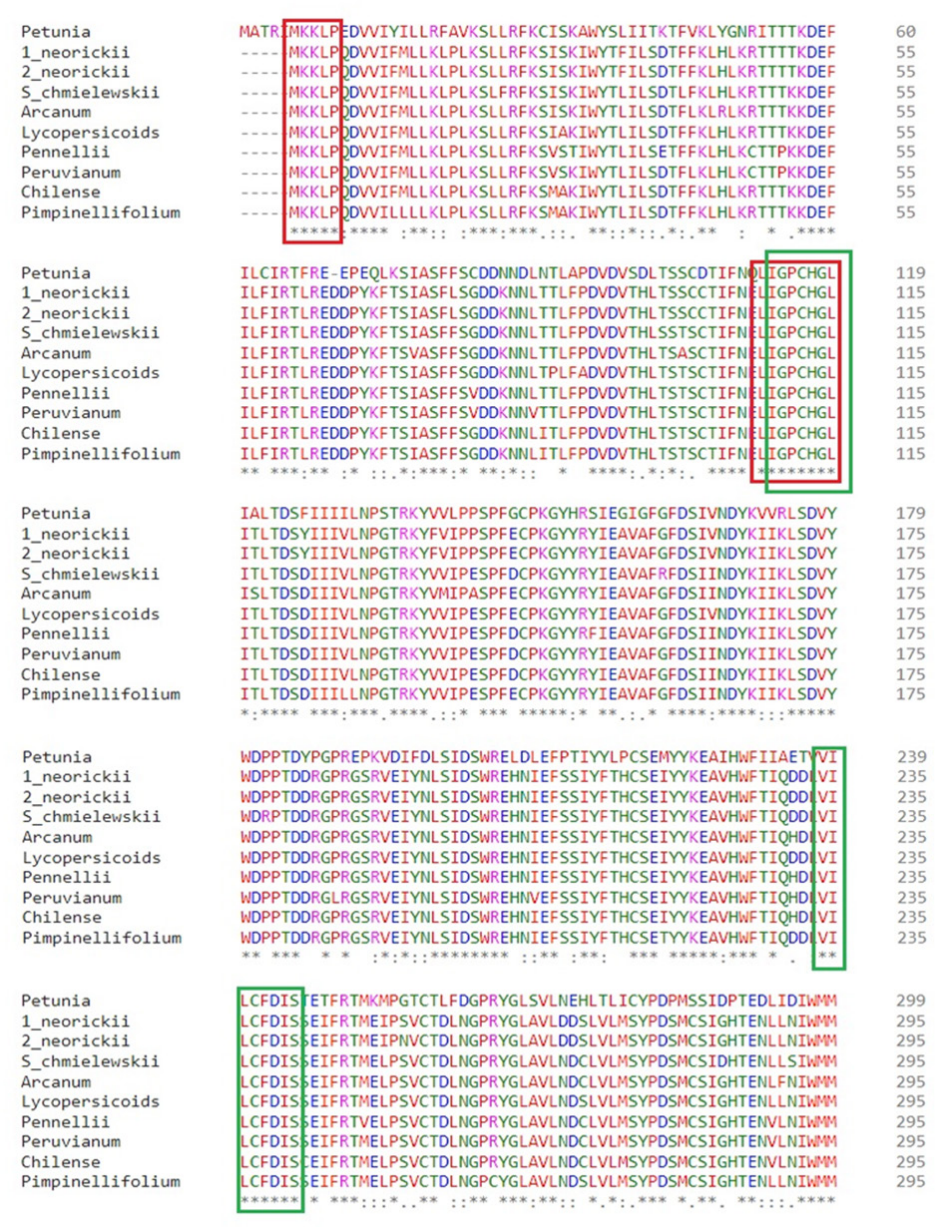
Multiple sequence alignment of S-locus F-box protein type-2 from *Petunia* with other *Solanum* SLFs. Red and green blocks highlight the highly conserved regions chosen to design forward and reverse primers for amplifying the first and second parts of the long amino acid sequence in *S. okadae*.

4. Scroll down the *multiple sequence alignment* result window to view the alignment. Target an area approximately 150–500 base pairs in length for optimal PCR amplification and position forward and reverse primers in more conserved regions.

5. Include between 5 and 7 amino acids in the primers, equating to ~15–20 base pairs (Figure 3).

6. To design a set of degenerate primers, convert the chosen amino acid sequence into the corresponding nucleotide sequence using the EMBOSS Backtranseq tool with *Solanum tuberosum* as a reference codon usage table.

7. Use the codes in Table 1 to represent the respective nucleotide(s) to design degenerate primers. For example, a primer with sequence 5′-AAWTTC-3′ will contain a mixture of 5′-AAATTC-3′ and 5′-AATTTC-3′ at equal concentrations (see General notes b, c, and d). Due to the inclusion of mixed bases required to capture sequence variability, degenerate primers often exhibit noticeable differences in melting temperature (T_m_) between forward and reverse pairs. This variation is a known feature of degenerate primer design and does not inherently compromise PCR performance.

*Note: The primers listed below in Table 2 are synthesized as degenerate primer mixtures based on the amino acid alignments and codon usage described in step B7. While degenerate nucleotide positions were used during synthesis—utilizing IUPAC ambiguity codes (see Table 1)—the sequences shown here represent one possible variant within each degenerate pool. These primers were designed to amplify putative SLF1, SLF2, and SLF3 of the S2 haplotype.*

**Table 1.** IUPAC ambiguity codes with respective complement

IUPAC code	Nucleotide	Complement
A	Adenine	T
G	Guanine	C
C	Cytosine	G
T	Thymine	A
Y	Pyrimidine (C/T)	R
R	Purine (A/G)	Y
W	Weak (A/T)	W
S	Strong (G/C)	S
K	Keto (T/G)	M
M	Amino (C/A)	K
D	A,G,T	H
V	A,C,G	B
H	A,C,T	D
B	C,G,T	V
X/N	Any base	X//N
-	Gap	-

**Table 2.** Degenerate primers for putative SLFs in *Solanum okadae*

Target	Primer	Sequence (5′–3′)	T_m_ (°C)	
**S_2_-SLF1**	OF1_F1	ATTCAATCTACTACTTTTATT	55
	OF1_R1	TCTAGTAGCTGGATTAAA	56
	OF2_F1	GCTACTAGAAATTTTAGA	56
	OF2_R1	CATAACCCAAACATGAAC	62
**S_2_-SLF2**	O2SLF2_F1	ATGAAGAAGGTTCCTCAAGAT	61
	O2SLF2_R1	AATAAGTCCATGACAAGGTCC	64
	O2SLF2_F2	ATTGGACCATGTCATGGACTT	59
	O2SLF2_R2	AGAAATATCAAAACAAAGAAT	54
**S_2_-SLF3**	O2SLF3_F1	AGATTTAAGTGTGTTACT	52
	O2SLF3_R1	AATTCTAACAACCTTATAAGT	55
	O2SLF3_F2	ACTTATAAGGTTGTTAGAATT	55
	O2SLF3_R2	AATAGCAAGTGGAGATTCAAT	61

*Note: In this experiment, despite some T_m_ divergence (up to 6 °C), the primers consistently produced specific amplicons of expected sizes under optimized conditions, confirming that amplification efficiency and specificity were not adversely affected.*

**C. Degenerate PCR**

1. In clean PCR tubes, set up a 20 μL reaction as shown in Table 3.

**Table 3.** PCR master mix components

Component	Amount
Sample DNA	10–50 ng
MyTaq reaction buffer (5×)	4 μL
Forward primer (10 µM)	1 μL
Reverse primer (10 µM)	1 μL
MyTaq DNA polymerase (5 U/µL)	0.5 μL
Water (dH_2_O)	up to 20 μL

*Note: In our experience, 10 ng of template DNA was sufficient. Use water as a negative control.*

2. Transfer the PCR tubes to an automated thermal cycler with the lid pre-heated to 105 °C and start the thermocycling procedure with the conditions shown in Table 4.

**Table 4.** PCR conditions used for amplification

Step	Temperature (°C)	Time	Cycles
Initial denaturation	94 ˚C	2 min	1
Denaturation	94 ˚C	30 s	35
Annealing*	(50–65) ˚C	30 s	
Extension	72 ˚C	1 min	
Final extension	72 ˚C	10 min	1
Hold	4 ˚C	∞	

*Annealing temperatures were optimized individually for each primer pair in this experiment.

**D. Gel purification and sequencing**

**Figure 4.**
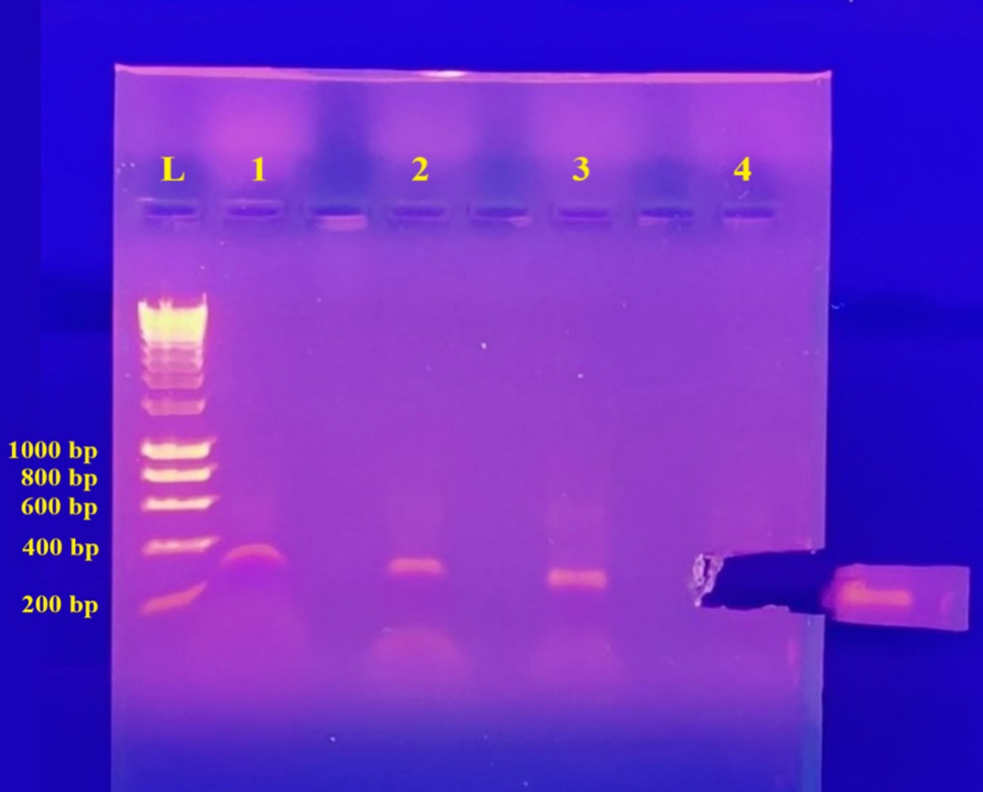
Retrieving the PCR product for purification using a UV transilluminator. Lane 1 (O2SLF2_F2 and O2SLF2_R2; 399 bp), lane 2 (O2SLF2_F1 and O2SLF2_R1; 342 bp), lane 3 (OF1_F1 and OF1_R1; 291 bp), and lane 4 (O2SLF3_F2 and O2SLF3_R2; 480 bp). Lane 4 was excised. Lane L: 1 kb DNA Hyper ladder. bp: base pair.

1. Prepare 0.8% (w/v) agarose gel with EtBr (see Recipe). Fill the casting tray with gel and allow it to set.

2. Immerse the gel completely in 1× TBE (running buffer).

3. Mix 10–12 μL of PCR reaction with 0.5–2.0 μL of DNA loading buffer blue (5× final concentration). Load into wells and run samples at 65–100 V for 60–75 min. Use 1 kb DNA HyperLadder as the reference.

4. Using a UV gel transilluminator, visualize the gel. Using a clean scalpel and forceps, cut the gel slice containing the DNA fragment. To reduce gel volume, cut as close to the DNA as possible (Figure 4). Weigh the gel slice in a pre-weighed 1.5 mL tube. Take note of the weight of the gel slice.

*Caution: Make sure to use appropriate protection while working with UV light and handling the gel containing EtBr.*

5. Using the GeneJET Gel Extraction kit, extract and purify the PCR product from the gel according to the manufacturer's instructions.

6. Sequence the PCR products (<800 bp) directly via Sanger sequencing using the SpeedREAD service provided by SourceBioscience (https://www.sourcebioscience.com/genomics/sanger-sequencing) with the same primers used for amplification. For amplicons over 800 bp, it is highly recommended to purify the PCR product using a cleanup kit (e.g., PCR cleanup column or gel extraction) to remove excess primers and dNTPs that could interfere with the sequencing reaction.

## Data analysis

**Figure 5.**
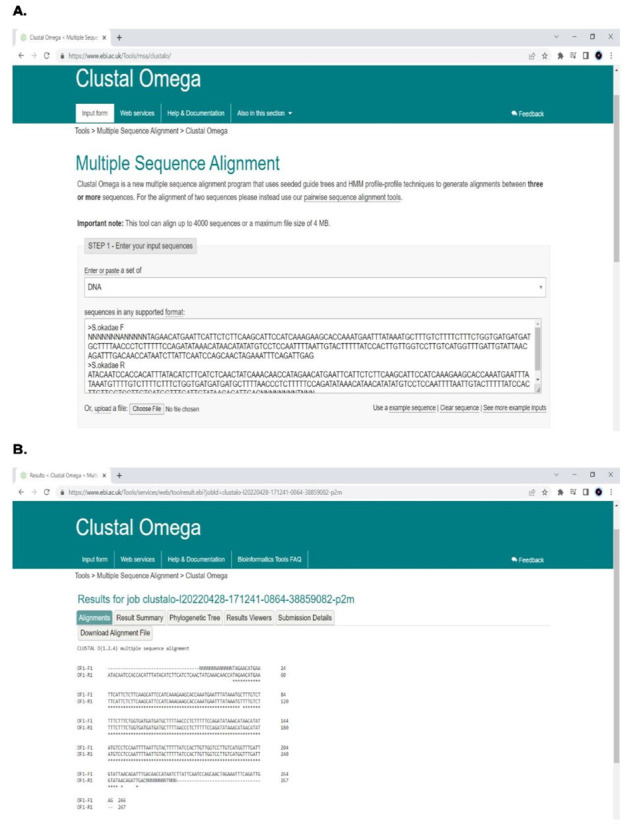
Nucleotide sequences subjected to alignment using ClustalΩ

1. Analyze the nucleotide sequences produced by PCR products using a sequence analysis tool such as SnapGene. Other tools like Benchling (https://www.benchling.com) or NCBI sequence analysis tools (https://www.ncbi.nlm.nih.gov/guide/sequence-analysis) can also be used.

2. Align the nucleotide sequences obtained post-sequencing (forward and reverse) using ClustalΩ (Figure 5).

*Note: To obtain an accurate sequencing of the full-length sequence, a minimum of two target sites should be sequenced in both directions with an overlap as depicted in Figure 3.*

**Figure 6.**
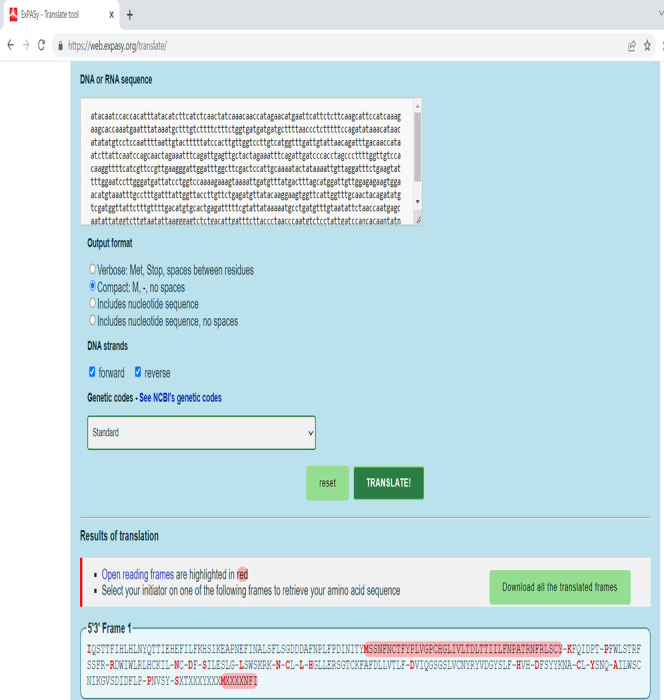
Translation of nucleotide sequence into the respective amino acid sequence using Expasy

**Figure 7.**
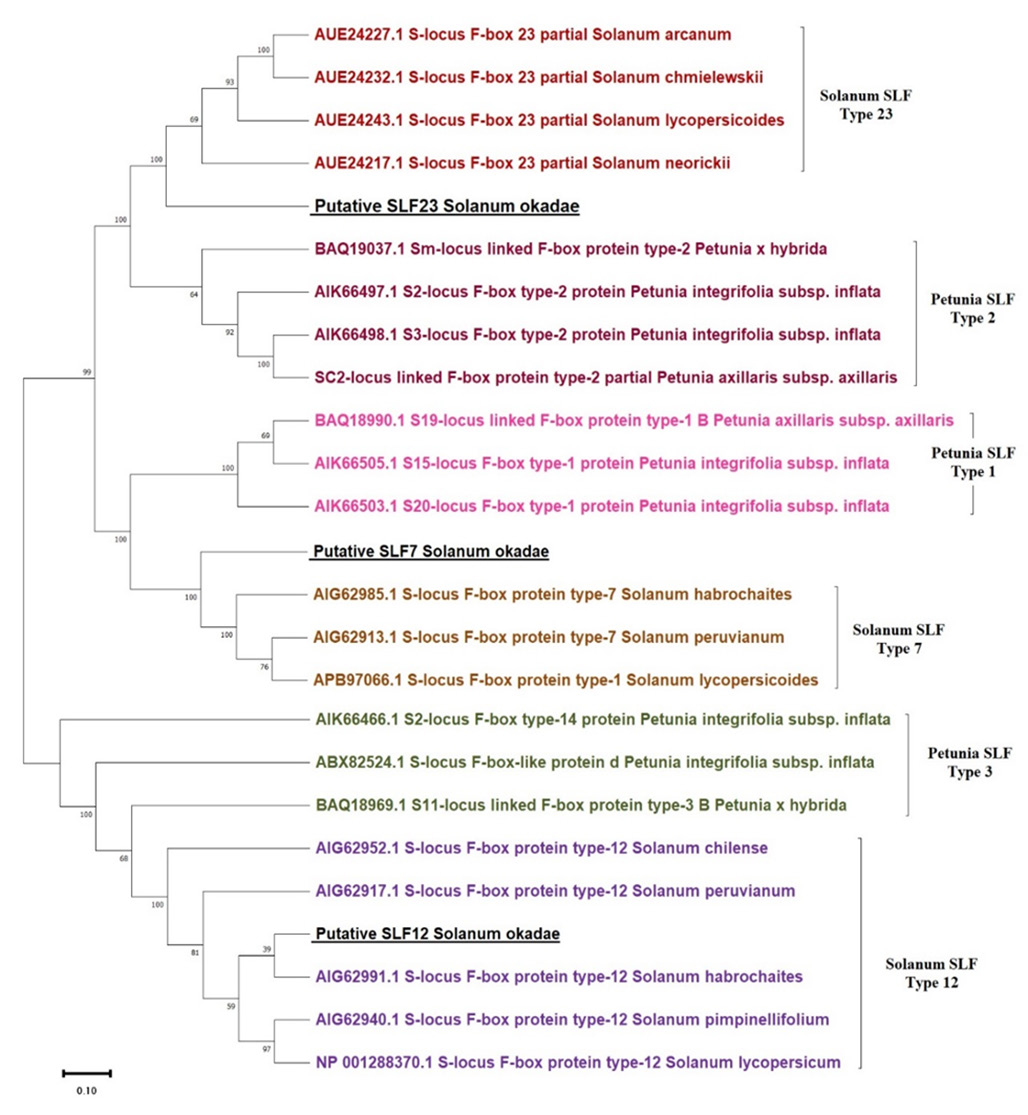
Phylogenetic tree for SLF-7, SLF-12, and SLF-23 from *Solanum* spp. and putative S-locus F-box proteins (SLFs) from *Solanum okadae* shown along with respective *Petunia* orthologs. The percentage of trees in which the associated taxa clustered together is shown next to the branches.

3. Once the sequences from the target sites are aligned, download the aligned sequences and join both sequences to get one complete nucleotide sequence.

4. Translate the joined nucleotide sequence into the corresponding amino acid sequence using the Expasy translation tool (Figure 6).

5. Perform a protein-protein BLAST alignment with the putative amino acid sequence as a query against the non-redundant protein sequence (nr) database and *Solanum* (taxid: 4107) as the organism.

6. On the *Result* window, click on *Multiple Alignment* to view the putative amino acid sequence aligned with similar amino acid sequences in closely related species.

7. Click *Distance tree of results* to see a dendrogram that groups sequences based on their distances from the query sequence. This display is useful for detecting aberrant or odd sequences in the BLAST output, as well as possibly natural groupings of related sequences, such as members of a gene family or homologs from other species.

8. A dendrogram/phylogenetic tree is presented in Figure 7 below. A more detailed description of the phylogenetic analysis can be found in chapter 7 of the study [5].

## Validation of protocol

This protocol was adapted from a previously established method [6] and successfully implemented in a comprehensive study focused on *S. okadae* [5]. Using this protocol, three distinct partial SLF sequences from *Solanum okadae* were amplified, sequenced, and validated through Sanger sequencing. The obtained sequences were deposited in the National Center for Biotechnology Information (NCBI) under accession numbers WHL25543, WHM28128, and WHM28129. These results confirm the reliability and reproducibility of the method for identifying SLF sequences in self-incompatible diploid potato.

Additional experimental evidence and data supporting the application of this protocol, including alignments and phylogenetic trees, are presented in Chapter 7 of the referenced doctoral thesis [5], which is publicly accessible via the University of Nottingham's research repository (https://eprints.nottingham.ac.uk/73456/). This serves as further validation of the protocol’s effectiveness and utility in related research.

## General notes and troubleshooting

**General notes**

1. For longer sequences (i.e., >800 bp), design the primers to include the start, middle, and end of the target sequence to get the complete sequence after sequencing. Keep the primer length between 18 and 25 nucleotides.

2. Avoid amino acids leucine, serine, and arginine, which may each be coded by six codon combinations, and try to include amino acids methionine and tryptophan, which are coded by a single codon.

3. Consider utilizing the base inosine (structurally identical to guanine), which may couple with any of the four bases but preferentially binds to cytosine if there is total degeneracy (no matches among any given species). To assure equimolar concentrations of each base at that place in your primer mix, substitute N for any base.

4. Degeneracy at the 3' terminal should be avoided (inosine should not be inserted here).

**Troubleshooting**

Problem 1: No distinct band appears in the PCR.

Possible cause: Primer issue or suboptimal PCR conditions.

Solution: Consider designing primers with enhanced target specificity or reduced degeneracy (<64-fold) and focus degenerate bases at the 5′ end, keeping the 3′ end specific. Excessive degeneracies can deteriorate primer concentration for any specific sequence. Consider minimizing degeneracy by employing a combination of primers derived from known sequences. A well-balanced level of primer degeneracy is crucial; too much degeneracy can lead to low specificity and reduced amplification efficiency, while too little may result in inadequate coverage of target sequence diversity. The ideal annealing temperature depends upon the primer sequences and is usually 2–5 °C below the lowest T_m_ of the pair. Start with a 50 °C annealing temperature and, if required, run a temperature gradient to identify the appropriate annealing temperature.

Problem 2: Preferential amplification of certain sequences.

Possible cause: Primer bias, sequence composition differences, or inefficient primer binding.

Solution: Adjust primer design to minimize extreme degeneracy. Use a mix of different primers targeting the conserved region. Optimize PCR conditions (e.g., Mg, dNTPs, annealing temperature).

Problem 3: Nonspecific amplification.

Possible cause: Degenerate primers can bind to unintended regions, especially in complex genomes or mixed samples.

Solution: Place degenerate bases away from the 3′ end (which is critical for extension). Add a GC clamp at the 3′ end for strong binding to the correct target. Perform in silico specificity checks (e.g., BLAST or Primer-BLAST).
